# Supplementary material for: A fault-intrusion-tolerant system and deadline-aware algorithm for scheduling scientific workflow in the cloud
Source: PeerJ Comput Sci. 2021 Nov 2;7:e747. doi: 10.7717/peerj-cs.747 (PMC8576554; doi:10.7717/peerj-cs.747)
Supplement: Supplemental Information 6 [file peerj-cs-07-747-s006.zip › Myproject1.1/docs/org/cloudbus/cloudsim/UtilizationModelStochastic.html]

UtilizationModelStochastic


JavaScript is disabled on your browser.


- Overview
- Package
- Class
- Use
- Tree
- Deprecated
- Index
- Help

- Prev Class
- Next Class

- Frames
- No Frames

- All Classes

- Summary:
- Nested |
- Field |
- Constr |
- Method

- Detail:
- Field |
- Constr |
- Method


org.cloudbus.cloudsim

## Class UtilizationModelStochastic

- java.lang.Object
- - org.cloudbus.cloudsim.UtilizationModelStochastic

- All Implemented Interfaces:
  :   UtilizationModel

  ---

    

  ```
  public class UtilizationModelStochastic
  extends java.lang.Object
  implements UtilizationModel
  ```

  The UtilizationModelStochastic class implements a model, according to which a Cloudlet generates
  random CPU utilization every time frame.

  Since:
  :   CloudSim Toolkit 2.0

- - ### Constructor Summary

    Constructors

    Constructor and Description

    `UtilizationModelStochastic()`

    Instantiates a new utilization model stochastic.

    `UtilizationModelStochastic(long seed)`

    Instantiates a new utilization model stochastic.
  - ### Method Summary

    Methods

    | Modifier and Type | Method and Description |
    | --- | --- |
    | `protected java.util.Map<java.lang.Double,java.lang.Double>` | `getHistory()` Gets the history. |
    | `java.util.Random` | `getRandomGenerator()` Gets the random generator. |
    | `double` | `getUtilization(double time)` Returns utilization in percents according to the time. |
    | `void` | `loadHistory(java.lang.String filename)` Load history. |
    | `void` | `saveHistory(java.lang.String filename)` Save history. |
    | `protected void` | `setHistory(java.util.Map<java.lang.Double,java.lang.Double> history)` Sets the history. |
    | `void` | `setRandomGenerator(java.util.Random randomGenerator)` Sets the random generator. |

    - ### Methods inherited from class java.lang.Object

      `clone, equals, finalize, getClass, hashCode, notify, notifyAll, toString, wait, wait, wait`

- - ### Constructor Detail


    - #### UtilizationModelStochastic

      ```
      public UtilizationModelStochastic()
      ```

      Instantiates a new utilization model stochastic.


    - #### UtilizationModelStochastic

      ```
      public UtilizationModelStochastic(long seed)
      ```

      Instantiates a new utilization model stochastic.

      Parameters:
      :   `seed` - the seed
  - ### Method Detail


    - #### getUtilization

      ```
      public double getUtilization(double time)
      ```

      **Description copied from interface: `UtilizationModel`**

      Returns utilization in percents according to the time.

      **Specified by:**
      :   `getUtilization` in interface `UtilizationModel`

      Parameters:
      :   `time` - the time

      Returns:
      :   utilization percentage


    - #### getHistory

      ```
      protected java.util.Map<java.lang.Double,java.lang.Double> getHistory()
      ```

      Gets the history.

      Returns:
      :   the history


    - #### setHistory

      ```
      protected void setHistory(java.util.Map<java.lang.Double,java.lang.Double> history)
      ```

      Sets the history.

      Parameters:
      :   `history` - the history


    - #### saveHistory

      ```
      public void saveHistory(java.lang.String filename)
                       throws java.lang.Exception
      ```

      Save history.

      Parameters:
      :   `filename` - the filename

      Throws:
      :   `java.lang.Exception` - the exception


    - #### loadHistory

      ```
      public void loadHistory(java.lang.String filename)
                       throws java.lang.Exception
      ```

      Load history.

      Parameters:
      :   `filename` - the filename

      Throws:
      :   `java.lang.Exception` - the exception


    - #### setRandomGenerator

      ```
      public void setRandomGenerator(java.util.Random randomGenerator)
      ```

      Sets the random generator.

      Parameters:
      :   `randomGenerator` - the new random generator


    - #### getRandomGenerator

      ```
      public java.util.Random getRandomGenerator()
      ```

      Gets the random generator.

      Returns:
      :   the random generator


- Overview
- Package
- Class
- Use
- Tree
- Deprecated
- Index
- Help

- Prev Class
- Next Class

- Frames
- No Frames

- All Classes

- Summary:
- Nested |
- Field |
- Constr |
- Method

- Detail:
- Field |
- Constr |
- Method
